# Modulation of Dextran Sodium Sulfate-Induced Colitis in Germ-Free Mice by *Enterococcus faecalis* Monocolonization

**DOI:** 10.3390/microorganisms13122864

**Published:** 2025-12-16

**Authors:** Beate Vestad, Petra Hanzely, Indrė Karaliūtė, Oda Ramberg, Jurgita Skiecevičienė, Rokas Lukoševičius, Jørgen V. Bjørnholt, Kristian Holm, Juozas Kupčinskas, Henrik Rasmussen, Johannes R. Hov, Espen Melum

**Affiliations:** 1 Norwegian PSC Research Center, Department of Transplantation Medicine, Division of Surgery and Specialized Medicine, Oslo University Hospital, 0424 Oslo, Norwayespen.melum@medisin.uio.no (E.M.); 2 Research Institute of Internal Medicine, Division of Surgery and Specialized Medicine, Oslo University Hospital, 0424 Oslo, Norway; 3 Institute of Clinical Medicine, Faculty of Medicine, University of Oslo, 0424 Oslo, Norway; 4 Department of Comparative Medicine, Division of Oslo Hospital Services, Oslo University Hospital, Rikshospitalet, 0424 Oslo, Norway; 5 Laboratory of Clinical and Molecular Gastroenterology, Institute for Digestive Research, Lithuanian University of Health Sciences, 44307 Kaunas, Lithuania; 6 Department of Gastroenterology, Lithuanian University of Health Sciences, 44307 Kaunas, Lithuania; 7 Department of Microbiology, Division of Laboratory Medicine, Oslo University Hospital, Rikshospitalet, 0424 Oslo, Norway; 8 Section of Gastroenterology, Department of Transplantation Medicine, Division of Surgery and Specialized Medicine, Oslo University Hospital, 0424 Oslo, Norway; 9 Hybrid Technology Hub-Centre of Excellence, Institute of Basic Medical Sciences, University of Oslo, 0424 Oslo, Norway

**Keywords:** inflammatory bowel disease (IBD), bacterial translocation, epithelial barrier dysfunction, host–microbe interactions, gut microbiota

## Abstract

Inflammatory bowel diseases (IBDs), including Crohn’s disease and ulcerative colitis (UC), are characterized by chronic gastrointestinal inflammation and involve complex interactions of genetic, environmental, and immune factors. *Enterococcus faecalis*, a gut commensal bacterium, has been implicated in IBD pathogenesis. This study investigated the effects of monocolonization with a UC-derived *E. faecalis* strain on acute dextran sulfate sodium (DSS)-induced colitis in germ-free (GF) mice, focusing on epithelial injury, inflammatory markers, hematologic indices, and bacterial translocation. In DSS-treated mice, monocolonization was associated with modest and mixed effects, including a higher colitis-related disease activity score, reduced anemia, increased fecal albumin and a trend towards reduced fecal calprotectin. Despite translocation of *E. faecalis* to mesenteric lymph nodes, no systemic dissemination was observed. Histological analysis revealed broadly similar inflammatory patterns between DSS-treated groups, with slightly more epithelial injury observed in colonized mice. These findings suggest that *E. faecalis* may influence discrete aspects of DSS injury in a strain-dependent and context-specific manner, rather than broadly altering overall disease severity. This study highlights the utility of GF models for examining strain-specific host–microbe interactions and underscores that individual bacterial isolates may exert heterogeneous and selective effects on acute colitis. Further research is needed to elucidate these complex mechanisms.

## 1. Introduction

Inflammatory bowel diseases (IBDs) encompass Crohn’s disease (CD) and ulcerative colitis (UC), both characterized by chronic gastrointestinal inflammation [1]. The pathogenesis of IBD involves a complex interplay of genetic susceptibility, environmental factors, and inappropriate immune activation [2]. This immune dysregulation includes abnormal activation of innate and adaptive immunity, with pro-inflammatory cytokines such as tumor necrosis factor-alpha (TNF-α) and interleukin (IL)-6 contributing to mucosal damage and perpetuating the inflammatory cycle [2,3,4]. Gut microbiota imbalance is also implicated, but the specific contributions of individual bacterial species to intestinal inflammation remain poorly understood [2,4].

Germ-free (GF) mice are invaluable tools for studying host–microbiota interactions, allowing researchers to isolate the effects of specific microbes or defined communities on disease processes [5,6]. In the dextran sulfate sodium (DSS)-induced colitis model, oral administration of DSS in drinking water induces severe colitis that mimics key features of human UC, including mucosal ulceration, epithelial barrier disruption, and inflammatory cell infiltration [7,8]. Mice undergoing DSS treatment typically exhibit weight loss, bloody diarrhea, and reduced activity, paralleling clinical symptoms of UC. While the precise mechanisms of DSS toxicity remain unclear, one hypothesis is that the sulphated, negatively charged DSS molecule interacts with dietary medium-chain fatty acids, forming complexes absorbed by colonic epithelial cells that contribute to barrier disruption and inflammation [9]. Other mechanisms, such as direct epithelial toxicity and activation of immune pathways, are also likely to play a role [9,10,11,12]. Unlike human UC, the development of DSS-induced colitis does not require adaptive immune cells, making this model particularly suitable for investigating the role of innate immune responses in intestinal inflammation [10]. The model is also useful for exploring microbial contributions to colitis progression, as the specificity of DSS to the colon is thought to depend on bacterial activity and local physiological factors such as water and electrolyte absorption [7,10].

Studying monocolonized mice provides a unique opportunity to investigate the effects of individual bacterial strains, helping to delineate their specific contributions to intestinal inflammation and immune responses [13]. *Enterococcus faecalis* (*E. faecalis*) is a Gram-positive gut commensal that can exhibit strain-dependent pathogenic properties and has been associated with intestinal inflammation in some IBD studies [14,15]. Several isolates demonstrate the ability to translocate across the intestinal barrier and reach lymph tissues, although the functional consequences of such strain-specific interactions remain incompletely understood. Several *E. faecalis* strains carry virulence-associated factors, including adhesins and hydrolytic enzymes, that may influence colonization dynamics or epithelial integrity [14,16,17,18,19]. Most experimental studies have assessed *E. faecalis* in conventional or chronic colitis models [20,21], and the strain-specific effects of monocolonization during acute DSS-induced injury under GF conditions remain insufficiently explored.

In the present study, we investigated how monocolonization with a UC-derived *E. faecalis* strain influences acute DSS-induced colitis in GF mice, focusing on predefined clinical, epithelial, inflammatory, and hematologic readouts.

## 2. Materials and Methods

### 2.1. Animals

Germ-free C57BL/6J mice, originating from the University of Bern Clean Mouse Facility, were bred in open cages (Eurostandard type II, 11bbB, Tecniplast, Buguggiate, Italy) maintained in sterile flexible-film isolators. At 5 weeks of age, the mice were earmarked and exported into an SPF facility in autoclaved GM500 individually ventilated cages (IVCs) (Tecniplast, Buguggiate, Italy) with bedding and nesting material. The animals were acclimatized for 7 days before they underwent monocolonization or remained GF as controls. GF status in the isolator was confirmed by monthly aerobic and anaerobic culture of fecal pellets and mold trap samples from the isolator, as well as yearly PCR-based serology testing according to FELASA recommendations [22]. In the IVCs, fecal pellets were cultured weekly during the experiment to monitor sterility and monoculture status of the animals. Group sizes were determined by the number of GF mice available for inclusion within a single experimental cycle.

The animals had ad libitum access to autoclaved chow pellets (LabDiet 5021, IPS Products Supplies, Alfreton, Great Britain) and water. Mice were kept in a humidity and temperature-controlled environment on a 12/12 h day/night cycle at an approved animal facility at the Oslo University Hospital, Rikshospitalet. The mice were segregated by sex and housed 2–3 mice in each cage during colonization, as well as before and during DSS administration. Cage change and experimental procedures were performed using sterile equipment by one sterile and one unsterile operator by strict aseptic handling of the cages inside a laminar air flow changing station [23]. At the end of the experiment, the mice were euthanized by heart puncture under isoflurane anesthesia followed by cervical dislocation and harvesting of organs.

### 2.2. Monocolonization

A single *Enterococcus faecalis* isolate used in this study was recovered from the mesenteric lymph nodes of a GF mouse after colonization with fecal material from an anonymized donor sample held in the Norwegian PSC (primary sclerosing cholangitis) Research Center biobank at Oslo University Hospital. The donor sample originated from an individual with a confirmed diagnosis of UC in clinical remission. Whole-genome sequencing of the isolate was performed, and annotated genomic data, including predicted virulence-associated and antimicrobial resistance genes, are provided in the Supplementary Material. *E. faecalis* was cultured on blood agar at 37 °C, 5% CO_2_ for 48–72 h. The bacterial stock solution was prepared by adding 20–30 middle big colonies into 1 mL of sterile PBS, equal to an optical density of 1.5–1.6 (2 × 10^9^ colony-forming units (CFU)/mL). An additional 3 mL PBS was added to make a final colonization solution of approximately 5 × 10^8^ CFU/mL. At 6 weeks of age, mice were colonized by slowly injecting 200 µL of the *E. faecalis* solution (10^8^ CFU) in a single dose via rectal administration using a gavage needle (18G) during manual restraint. After 21 days, a subset of the colonized mice was subjected to DSS administered in autoclaved drinking water for 7 days. Noncolonized GF mice with DSS administration served as colitis controls, whereas colonized GF mice without DSS served as colonization controls.

### 2.3. DSS Colitis Model

Dextran sulfate sodium (Cat no. DB001, Batch no. DB001-47, 35,866 kDa MW, TdB Labs AB, Uppsala, Sweden) was administered to 9-week-old mice at 2.5% concentration (*w*/*v*) in autoclaved drinking water for 7 days to induce acute colitis. The start of DSS dosing was defined as Day 0. During DSS administration, the mice were monitored daily with measurements of food and water intake, body weight, and scoring according to disease activity. Mice were euthanized according to humane endpoint (HEP) scoring in cases of weight loss above 20% from DSS start, severe rectal prolapse, isolated behavior or reaching HEP score of 14 or higher (Supplementary Table S1). DSS-induced colitis development was evaluated by calculating a separate Disease Activity Index (DAI) score based on weight loss, stool consistency, and rectal bleeding (modified from [24,25]). The DAI was calculated as the average of the total scores consisting of: weight loss (0: 0–5%; 1: 6–10%; 2: 11–15%; 3: 16–20%; 4: >20%), stool consistency (0: none; 2: loose stools; 4: gross diarrhea), and rectal bleeding (0: normal; 2: mild bleeding, visible in stool; 3: moderate bleeding, visible from rectum and/or in bedding/cage; 4: gross bleeding, marked staining in bedding/cage). Body weight development during DSS exposure and colon length at harvest were predefined as primary outcomes in the evaluation of DSS-induced colitis. Secondary outcomes included the DAI score, histological scores, fecal biomarkers, hematologic indices, and colonization/translocation measures.

### 2.4. Sample Collection and Processing

Fresh fecal samples were collected before and after DSS administration for analysis of albumin and calprotectin and kept on ice until storage at −20 °C. Blood was collected by cardiac puncture under isoflurane anesthesia into a syringe coated with 0.5 M EDTA using a 23 G needle after sterilizing the insertion site with 70% ethanol. Mesenteric lymph nodes were collected by aseptic technique and cultured to monitor for bacterial translocation. Briefly, 2–3 lymph nodes were homogenized in 100–200 µL PBS, and 100 µL of the homogenate was cultured on blood agar plates overnight at 37 °C, 5% CO_2_. Subsequently, liver, spleen and cecum were collected and weighed. Colon length was measured before the entire colon was processed for histology assessment by “swiss roll” preparation on a 27 G needle. Fecal pellets and cecal content for DNA extraction were snap frozen on dry ice and kept at −80 °C. Blood plasma was prepared by centrifugation of whole blood twice at 2500× *g* for 15 min.

### 2.5. Hematological Parameters

Determination of hematological parameters was performed in 10 µL fresh EDTA-blood using an ABX Micros 60 automated hematology instrument (Horiba ABX SAS, Montpellier, France).

### 2.6. Histology

Tissue sections were fixed in 4% PFA for 18 h and placed in cold PBS, then paraffin-embedded, cut, and stained with hematoxylin and eosin (H&E). Two different sections were evaluated per sample, taken 15 µm apart. Sections were scanned using Olympus SLIDEVIEW™ VS200 Slide scanner (Olympus Corporation, Tokyo, Japan) and images processed using QuPath 0.4.3 software [26]. The images were examined and scored for signs of colitis by one of the authors (B.V.), blinded to group allocations. The colon sections were divided into three main scoring regions: proximal colon, middle colon and distal colon. Histology assessment of the following parameters; I: mononuclear cell infiltration, II: polymorphonuclear cell infiltration, III: epithelial hyperplasia, as well as IV: epithelial injury, comprising visible erosion, ulcerations and alterations to the crypt architecture, were scored for each region of the colon as absent (0), mild (1), moderate (2), or severe (3) [27,28]. A total colon score was created by calculating an average of the three colonic region scores.

### 2.7. Albumin and Calprotectin Measurements

Fecal albumin was analyzed using Mouse Albumin ELISA Kit (Cat E99-134, Bethyl Laboratories, Montgomery, TX, USA) and fecal calprotectin was analyzed using the S100A8/S100A9 ELISA Kit (Ref. KR6936, Immundiagnostik AG, Bensheim, Germany), according to the manufacturer’s recommendations. Briefly, fecal pellets were diluted 1:10 with dilution buffer from the albumin kit, placed on ice for 15 min and thoroughly homogenized before centrifugation at 13,000 RPM for 5 min at 4 °C. The supernatant was collected and used for albumin measurements. The remainder of the sample material was further diluted 1:5 with extraction buffer from the calprotectin kit and centrifugated at 2000× *g* for 10 min at 4 °C. The supernatant was collected and used for calprotectin measurements. Albumin and calprotectin results were obtained using a BioTek Synergy H1 Hybrid plate reader (Agilent, Santa Clara, CA, USA).

### 2.8. DNA Extraction and Real-Time Quantitative PCR of E. faecalis

DNA was extracted from one pellet of mouse feces (20–60 mg) or 200 µL of cecal content per mouse using the Genesig^®^ Easy DNA/RNA extraction Kit (Primerdesign™ Ltd., Eastleigh, UK) and mouse plasma DNA was extracted using the QIAamp MinElute ccf DNA Kit (Cat 55284, Qiagen, Hilden, Germany), following the manufacturer’s recommendations. A template DNA input of 1 ng was used per reaction, brought to a final volume of 8 µL with DNase/RNase-free H_2_O. Copy numbers of *E. faecalis* DNA were then quantified using the *Enterococcus faecalis* qPCR Test Kit (YouSeq Ltd., Winchester, United Kingdom) and a Stratagene Mx3000P real-time PCR cycler and MxPro software v4.10d (Agilent). The PCR cycling conditions used were: 3 min hot start at 95 °C followed by 45 cycles of 15 s at 95 °C and 60 s at 60 °C. Fluorogenic data was collected both through the FAM (*E. faecalis*/sample) and HEX (supplied endogenous control) channels.

### 2.9. Statistical Analysis

Normality of data distributions was assessed using the Shapiro–Wilk test. For comparisons between two groups, normally distributed data were analyzed using two-tailed unpaired *t*-tests, whereas non-parametric data were analyzed using two-tailed Mann–Whitney *U* tests. Comparisons across three or more groups were performed using one-way ANOVA with the Bonferroni correction for multiple comparisons or Kruskal–Wallis tests with Dunn’s post hoc test when appropriate. Body weight at selected time points was compared using a two-tailed unpaired *t*-test. DAI score trajectories were analyzed using two-way ANOVA with group and time as factors, using the software’s default correction for repeated-measures data. A *p*-value < 0.05 was considered statistically significant. All statistical analyses were performed using GraphPad Prism version 10.2.0 (Boston, MA, USA).

## 3. Results

### 3.1. E. faecalis Monocolonization and Colitis Induction

**Figure 1:**
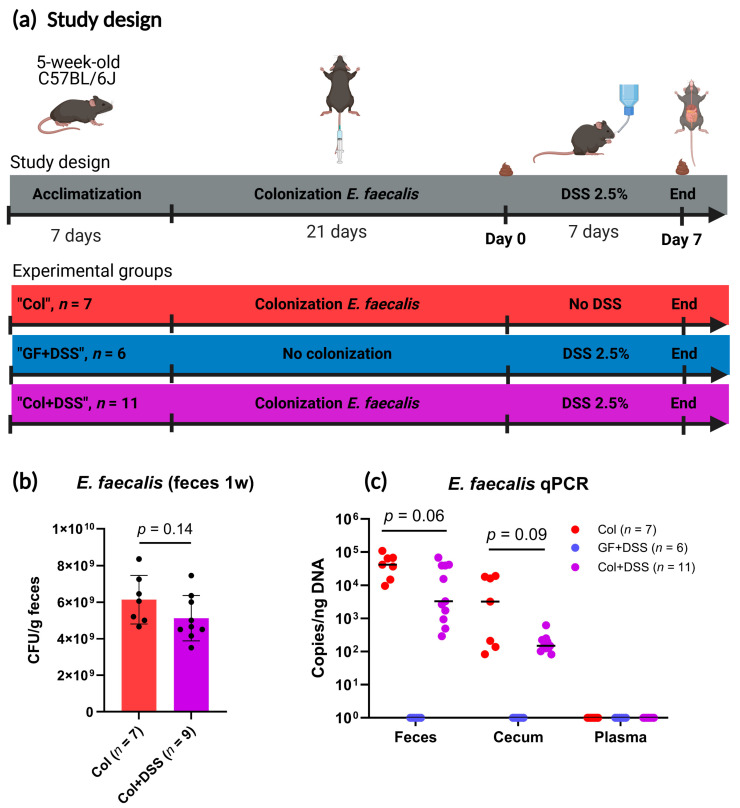
Study design and validation of colonization. (**a**) Overview of the study design and allocation of experimental groups. (**b**) Culture-based detection of *E. faecalis* in mouse fecal samples one week after colonization. Individual values are shown with mean ± SD, statistical comparison performed using a two-tailed unpaired *t*-test. (**c**) qPCR quantification of *E. faecalis* DNA (log10 copies per ng total DNA) in feces, cecum and plasma. Lines indicate median values. Fecal samples from colonized mice with and without DSS treatment were compared using the Mann–Whitney *U* test.

A total of 24 GF C57BL/6J mice (16 males and 8 females) were included in the study. To assess the effect of *E. faecalis* on colitis development, mice were monocolonized with *E. faecalis* 21 days (3 weeks) prior to administration of autoclaved drinking water with DSS (*n* = 11, 6 males and 5 females), or autoclaved water only (*n* = 7, 4 females and 3 males) for 7 days. Sterile water with DSS was provided to a control group of GF mice (*n* = 6 males) (Figure 1a). The DSS dosage applied was based on titration in a separate pilot study to ensure proper colitis development without reaching unacceptable toxicity (Supplementary Material). At the end of the experiment (day 7 after DSS start, day 35 after acclimatization start), the mice were euthanized and organs harvested. To confirm successful monocolonization or GF status, fresh fecal samples were collected one week after colonization. As expected, *E. faecalis* colonies were detected in fecal pellets from all colonized mice (Figure 1b). To further validate colonization and assess potential bacterial translocation, we quantified *E. faecalis* DNA in feces, cecum, and plasma collected at euthanasia. In fecal samples, the median load was ~40,000 copies per ng total DNA in colonized mice without DSS, with a clear trend toward lower copy numbers in DSS-treated colonized mice (*p* = 0.06) (Figure 1c). *E. faecalis* was also consistently detected in all cecum content samples from colonized mice, although at substantially lower copy numbers (median ~3000 copies for colonized mice and ~150 copies for DSS-treated colonized mice). No *E. faecalis* DNA was detected in plasma from any mouse group, indicating that circulating *E. faecalis* DNA levels, if present, were below the detection limit of the assay. As expected, no *E. faecalis* DNA was detected in samples from the GF mice receiving DSS (Figure 1c). Whole-genome sequencing of the isolate confirmed the presence of multiple virulence- and resistance-associated genetic features, as detailed in the Supplementary Material, providing genomic context for the strain used in this model.

### 3.2. E. faecalis-Colonization Alters Colitis-Related Disease Activity and Physiological Parameters in DSS-Treated Mice

**Figure 2:**
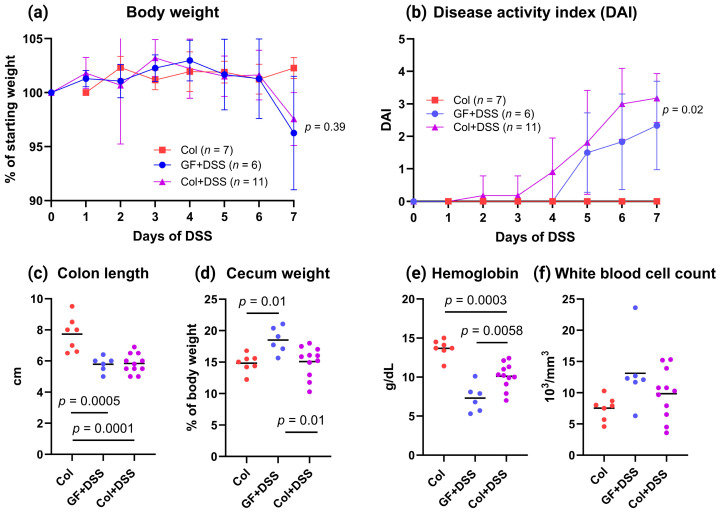
Disease activity measures during DSS exposure and endpoint assessments at Day 7. (**a**) Body weight expressed as percentage of starting weight, shown as mean ± SD. Comparison between the two DSS-treated groups at Day 7 was performed using a two-tailed unpaired *t*-test. (**b**) Disease Activity Index (DAI), incorporating weight loss, stool consistency, and rectal bleeding, shown as mean ± SD. Differences between the two DSS-treated groups across the time course were assessed using two-way ANOVA (group effect). (**c**) Colon length in cm. (**d**) Cecum weight expressed as a percentage of body weight. (**e**) Hemoglobin concentration. (**f**) White blood cell count. Panels (**c**–**f**) show individual values with the mean indicated by a line. Statistical comparisons were performed using one-way ANOVA with the Bonferroni correction for multiple comparisons; only significant adjusted *p*-values are shown.

The mice in both DSS-treated groups experienced a clear weight loss on Day 7 of DSS administration, without a significant difference between the *E. faecalis*-colonized mice and the GF mice at harvest (Figure 2a). The colitis-related DAI score increased over time in both DSS-treated groups, with overall significantly higher scores in mice pre-colonized with *E. faecalis* compared to GF mice (two-way ANOVA, group effect, *p* = 0.02) (Figure 2b). Both DSS groups had shorter colons compared to colonized mice only (*p* < 0.001), but with no difference between the DSS groups (Figure 2c). Mean cecum weight in GF mice receiving DSS was 18.5% of body weight, and it was lower in both colonized groups, although significantly reduced only in colonized mice receiving DSS (Figure 2d). Both DSS groups had a higher degree of anemia compared to the mice that were only *E. faecalis*-colonized, while the GF mice treated with DSS had lower blood hemoglobin than colonized mice treated with DSS (Figure 2e). No changes were observed between the groups in levels of circulating white blood cells (Figure 2f).

### 3.3. DSS Treatment of GF and E. faecalis-Monocolonized Mice Induces Similar Histology Alterations

**Figure 3:**
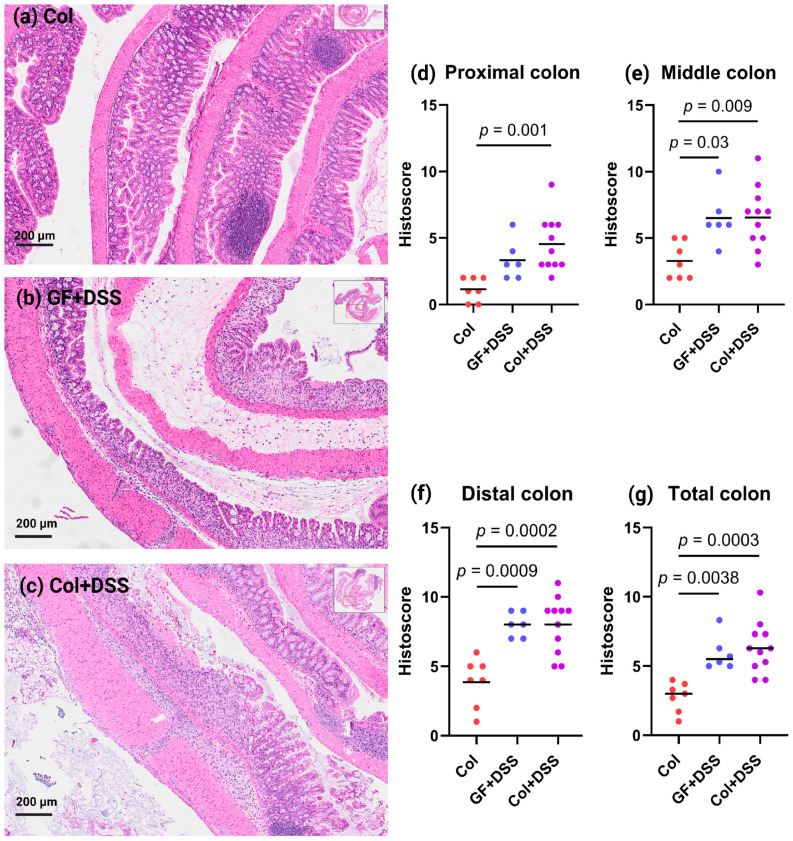
Histological alterations following DSS treatment in GF and monocolonized mice. Representative H&E-stained colon sections from (**a**) an *E. faecalis*-colonized mouse without DSS, (**b**) a GF mouse treated with DSS, and (**c**) an *E. faecalis*-colonized mouse treated with DSS. Histological scores for (**d**) proximal, (**e**) middle, (**f**) distal, and (**g**) total colon (mean of the three regions) are shown as individual values with the mean indicated by a line. Statistical comparisons were performed using one-way ANOVA with the Bonferroni correction for multiple comparisons; only adjusted *p*-values < 0.05 are shown. H&E: hematoxylin and eosin.

In mice colonized with *E. faecalis* without DSS treatment, we observed mild to moderate hyperplasia and a few immune cell aggregates. Some of the mice had mild edema and erosion in the mucosa, while the epithelium was generally intact (Figure 3a). GF mice treated with DSS displayed histological features compatible with loss of crypts, erosion, edema and infiltrations of immune cells into muscle layers (Figure 3b). Similarly, *E. faecalis*-colonized mice with DSS-induced colitis showed comparable histological alterations, although with somewhat more pronounced ulcerations and damaged crypt architecture (Figure 3c).

Histological scoring of the different colonic regions revealed that inflammation and epithelial injury were more pronounced in the middle and distal colon than in the proximal colon (Figure 3d–f). There were no statistical differences between the two groups of DSS-treated mice for any of the colonic regions. However, comparing the two groups of colonized mice, DSS-treated mice had higher histoscores than non-DSS-treated mice (Figure 3d–g).

### 3.4. E. faecalis Alters Fecal Calprotectin and Albumin Levels and Translocates to Mesenteric Lymph Nodes

**Figure 4:**
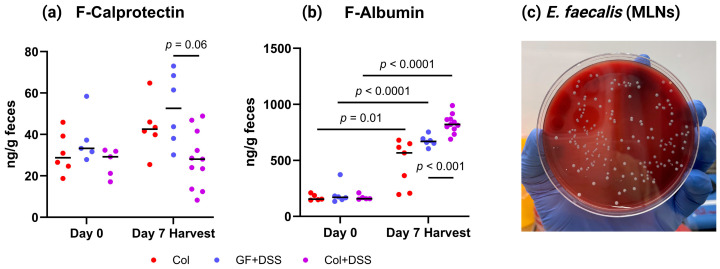
Markers of intestinal inflammation and bacterial translocation. (**a**) Fecal calprotectin concentrations (ng/g feces) measured at Day 0 (Col: *n* = 6, GF + DSS: *n* = 5, Col + DSS: *n* = 5) and Day 7 (Col: *n* = 6, GF + DSS: *n* = 6, Col + DSS: *n* = 11). (**b**) Fecal albumin concentrations (ng/g feces) at Day 0 (Col: *n* = 5, GF + DSS: *n* = 6, Col + DSS: *n* = 5) and Day 7 (Col: *n* = 7, GF + DSS: *n* = 6, Col + DSS: *n* = 11). Individual values are shown with mean ± SD. Statistical comparisons at each time point were performed using two-tailed unpaired *t*-tests. (**c**) Representative overnight culture of mesenteric lymph nodes (MLNs) from an *E. faecalis*-colonized mouse collected at the end of the experiment. CFU: colony-forming unit.

Levels of the intestinal inflammation marker calprotectin, measured before and after DSS treatment, did not differ significantly between colonized and non-colonized mice (Figure 4a). However, at Day 7, levels were lower in colonized DSS-treated mice than in GF DSS-treated mice, but this difference was not statistically significant (Figure 4a). Moreover, fecal albumin levels increased significantly from Day 0 to Day 7 in all DSS-treated mice, with the highest levels observed in colonized mice receiving DSS (Figure 4b).

As fecal albumin may reflect intestinal barrier leakage and *E. faecalis* has been reported to translocate to lymph nodes in mice [29], we examined mesenteric lymph nodes (MLNs) from a subset of colonized animals (*n* = 9). Overnight aerobic cultures confirmed viable *E. faecalis* in MLNs from all colonized mice, regardless of DSS exposure (representative culture shown in Figure 4c). Quantitative colony counts showed numerically higher values in colonized mice without DSS than in the Col + DSS group, although this difference was not statistically significant (Mann–Whitney *U* test, *p* = 0.1) (Supplementary Figure S8).

## 4. Discussion

Growing evidence suggests that manipulation of the intestinal microbiota can influence the development and progression of chronic inflammatory diseases such as IBD [30,31,32]. In this study, we used GF mice and a chemically induced colitis model to investigate the effect of monocolonization using an *E. faecalis* strain isolated from a patient with UC. We found that 3 weeks of monocolonization (using a single rectal dose of 10^8^ CFU) followed by 7 days of 2.5% DSS administration resulted in modest and heterogeneous effects on several clinical, inflammatory, and epithelial readouts. Specifically, *E. faecalis* monocolonization led to a higher colitis-related disease activity (DAI score), reduced anemia, increased fecal albumin levels, and trends towards lowered fecal calprotectin compared to GF mice receiving DSS alone. No significant differences were observed in body weight or colon length, and the measurable changes were largely confined to selected secondary readouts, supporting the interpretation that this strain exerted a limited and variable overall impact on DSS-induced colitis. Moreover, exploratory sex-stratified analyses did not reveal consistent sex-related differences in body weight responses (Supplementary Figure S7).

Early fecal detection and endpoint identification of *E. faecalis* in both cecum and mesenteric lymph nodes indicate that the strain could cross the epithelial barrier under the conditions of this model. The markedly lower cecal copy numbers likely reflect dilution by host-derived DNA, rather than reduced bacterial presence, and should therefore be interpreted cautiously.

The DSS-induced colitis model in experimental mice is well-documented and mimics key features of human UC, including rectal bleeding, diarrhea, and weight loss [8,10]. In line with these expected disease traits, mice in the present study exhibited shortened colons and weight loss towards the end of the experiment. Although weight loss did not differ between the DSS-treated groups, *E. faecalis*-colonized mice displayed higher DAI scores, indicating that this strain influenced selected clinical features of DSS-induced injury. The reduced anemia in colonized mice may reflect differences in the dynamics of blood loss or epithelial damage, although alternative explanations are equally plausible. This aligns with previous reports showing that DSS treatment in GF mice can lead to substantial blood loss, often used as an indicator of disease severity [33,34].

Monocolonization studies using probiotic or disease-associated bacteria have been shown to affect the severity of chemically induced colitis in GF mice. For example, colonization with *Bacteroides fragilis* has been demonstrated to protect against DSS-induced acute colitis by increasing survival and reducing immune cell infiltration and colon shortening [35,36]. In contrast, certain microbes have been suggested to exacerbate colitis development in chemically induced models. For example, mucosa-associated microbes from patients with UC have been shown to increase susceptibility to DSS-induced colitis in GF BALB/c mice, although they did not induce spontaneous colitis on their own [37]. Additionally, strains of *E. faecalis* isolated from the inflamed mucosa of UC patients are highly adherent and are likely to carry virulence-related genes, contributing to disease activity [38]. In GF mouse models of chronic colitis, *E. faecalis* monocolonization has been shown to primarily induce colitis in the distal colon [15,39]. Our findings showed a comparable pattern, with higher histoscores in the middle and distal colon relative to the proximal region in both *E. faecalis*-colonized mice (with and without DSS treatment) and in GF mice. Previous studies have demonstrated that *E. faecalis* can form a uniform biofilm in the GF murine gut without inducing pronounced inflammation [40], and our observations are consistent with this. The combined biomarker and histological findings in our study point toward a complex and multidirectional injury pattern. Reduced anemia and numerically lower fecal calprotectin levels suggest attenuation of certain inflammatory or bleeding-related components of DSS injury, whereas increased albumin and a tendency toward mucosal erosion indicate greater epithelial compromise. Rather than supporting a single directional effect, these findings imply that the clinical isolate used here modulated distinct epithelial and inflammatory endpoints in different ways. Such a pattern may reflect temporal differences in how DSS injury unfolds in colonized versus GF mice, variation in immune cell recruitment dynamics, or differential sensitivity of the biomarkers to early versus late injury phases.

Colonization of GF mice with monocultures like *E. faecalis* prior to the chemical induction of acute colitis may have several effects on colitis development [15]. One possible explanation is variation in the dynamics of epithelial injury and innate immune responses, rather than a uniform increase in susceptibility. We found that mice colonized with *E. faecalis* had numerically lower fecal calprotectin levels compared to GF mice treated with DSS. In general, increased fecal calprotectin reflects neutrophil infiltration in the gut, corresponding with active inflammation. While direct studies on fecal calprotectin in *E. faecalis*-monocolonized GF mice are scarce, existing research on gut inflammation markers suggests that fecal calprotectin typically increases under inflammatory conditions [41]. The lower values observed in our study are therefore compatible with strain-dependent or context-specific host–microbe interactions, but mechanistic inference is limited. Several alternative explanations are possible. Strain-specific differences in *E. faecalis* virulence factors could influence host immune responses via multiple pathways. For example, some strains may modulate the immune system by inducing production of anti-inflammatory cytokines such as IL-10 [42,43], potentially reducing neutrophil recruitment and activation. Additionally, certain *E. faecalis* strains have been reported to reduce expression of pro-inflammatory cytokines such as TNF-α and IL-6, which drive neutrophil-mediated inflammation in colitis models [44]. Such effects may involve interactions with innate immune signaling pathways, but the present study did not assess these mechanisms directly, and mechanistic interpretations remain speculative. Probiotic *E. faecalis* strains have also been reported to enhance mucosal integrity and reduce epithelial damage in colitis models [45,46,47]. However, most studies have been conducted in conventional hosts, and responses in gnotobiotic systems may differ, as Enterococcus species can provoke stronger inflammatory reactions in disease-susceptible hosts such as IL-10 knockout mice [48,49]. Importantly, the strain used in this study carries several putative virulence-associated genes, including adhesins, surface-binding proteins, degradative enzymes, and aggregation substance proteins, which could promote close epithelial interaction or localized tissue disruption. Such genomic features may help contextualize the observed increase in fecal albumin and the trend toward epithelial erosion, while the numerical reduction in calprotectin underscores that these effects do not necessarily co-occur with heightened neutrophil-driven inflammation. Collectively, these findings emphasize that *E. faecalis* may shape DSS-induced injury along multiple, potentially opposing dimensions, consistent with strain-specific and non-uniform effects on host responses.

As with all germ-free DSS models, several system-specific factors also influence how these results should be interpreted. The lack of a conventional microbiota group limits comparison to a more physiologic inflammatory context, and the acute, single-time-point design does not capture potential longer-term or relapsing patterns of disease. These features define the experimental setting in which the present findings were generated and help delineate the aspects of host–microbe interaction that can be inferred from this model.

## 5. Conclusions

In summary, monocolonization of GF mice with *E. faecalis* prior to DSS-induced acute colitis influenced selected clinical, epithelial and inflammatory features of DSS-induced injury, including a higher colitis-related disease activity score, together with modest and variable differences across histological and molecular readouts. A key strength of this study is the use of GF mice and a controlled DSS model, which allows focused examination of how a single bacterial isolate shapes host epithelial and inflammatory responses. While our work captures only a portion of the complex interplay between host immunity, microbial colonization, and DSS-induced injury, it demonstrates that microbial effects can be context-dependent and act on selected components of intestinal damage. Importantly, because only one clinical *E. faecalis* isolate was examined, these findings should not be generalized to the species level. Comparative studies including strains from healthy donors and diverse IBD phenotypes are needed to determine the range of strain-dependent effects and further elucidate their contribution to inflammatory responses and epithelial injury in colitis models.

## Data Availability

Other data generated during the study are available from the corresponding author upon reasonable request.

## Supplementary Materials

The following supporting information can be downloaded at: https://www.mdpi.com/article/10.3390/microorganisms13122864/s1. https://doi.org/10.5281/zenodo.17829174, Supplementary Material: ARRIVE checklist and raw numerical endpoint data underlying the data presented in the manuscript and figures (published 14 March 2025; version v2 updated 5 December 2025); NCBI Sequence Read Archive (SRA): Raw sequencing reads for *Enterococcus faecalis* under accession number SRR32735232, linked to BioProject PRJNA1236225; https://doi.org/10.5281/zenodo.15023961, Supplementary Data: Gene predictions and typing results from RVFScan, CARD scan, and PubMLST (published 14 March 2025).

## Abbreviations

The following abbreviations are used in this manuscript:

IBD	Inflammatory bowel disease
*E. faecalis*	*Enterococcus faecalis*
GF	Germ-free
DSS	Dextran sulfate sodium
MLNs	Mesenteric lymph nodes
UC	Ulcerative colitis
TNF-α	Tumor necrosis factor alpha
IL	Interleukin
CFU	Colony-forming units
