# Supplementary material for: Modulation of Dextran Sodium Sulfate-Induced Colitis in Germ-Free Mice by Enterococcus faecalis Monocolonization
Source: Microorganisms. 2025 Dec 16;13(12):2864. doi: 10.3390/microorganisms13122864 (PMC12735899; doi:10.3390/microorganisms13122864)
Supplement: Supplementary file 1 [file microorganisms-13-02864-s001.zip › microorganisms-3995189-supplementary/Supplementary Material_microorganisms.pdf]

Supplementary Material

Modulation of dextran sodium sulfate-induced colitis in germ-free mice by *Enterococcus faecalis*-monocolonization

Beate Vestad[1,2,3], Petra Hanzely[1,2,3], Indrė Karaliūtė[4], Oda Ramberg[1,2], Jurgita Skiecevičienė[4,5], Rokas Lukoševičius[4], Jørgen V. Bjørnholt[3,6], Kristian Holm[1,2,3], Juozas Kupčinskas[4,5], Henrik Rasmussen[3,7], Johannes R Hov[1,2,3,8], Espen Melum[1,2,3,8,9]

Affiliations

1 Norwegian PSC Research Center, Department of Transplantation Medicine, Division of Surgery and Specialized Medicine, Oslo University Hospital, Oslo, Norway.

2 Research Institute of Internal Medicine, Division of Surgery and Specialized Medicine, Oslo University Hospital, Oslo, Norway.

3 Institute of Clinical Medicine, Faculty of Medicine, University of Oslo, Oslo, Norway.

4 Laboratory of Clinical and Molecular Gastroenterology, Institute for Digestive Research, Lithuanian University of Health Sciences, Kaunas, Lithuania.

5 Department of Gastroenterology, Lithuanian University of Health Sciences, Kaunas, Lithuania.

6 Department of Microbiology, Division of Laboratory Medicine, Oslo University Hospital, Rikshospitalet, Oslo, Norway.

7 Department of Comparative Medicine, Division of Oslo Hospital Services, Oslo University Hospital, Rikshospitalet, Oslo, Norway.

8 Section of Gastroenterology, Department of Transplantation Medicine, Division of Surgery and Specialized Medicine, Oslo University Hospital, Oslo, Norway.

9 Hybrid Technology Hub-Centre of Excellence, Institute of Basic Medical Sciences, University of Oslo, Oslo, Norway.

Genomic properties of the *Enterococcus faecalis* strain used for monocolonization

To investigate the genomic properties of the *E. faecalis* strain used in the study, we performed whole-genome sequencing of the bacterial isolate. Results from the gene prediction analyses can be accessed at https://doi.org/10.5281/zenodo.15023961. Briefly, the strain was identified as type ST124,

determined by multilocus sequence typing (MLST) (https://pubmlst.org/). To investigate potential virulence factors contributing to pathogenic properties, we used RVFscan virulence database (https://rvfscan.hugobiotech.com/task/index). Thirty-one genes encoding for virulence factors, including fibrinogen-binding proteins, degradative enzymes, capsule proteins and aggregation substance proteins were found. Notable genes include *fss1* and *fss2* (fibrinogen-binding proteins), *EF3023* and *EF0818* (hyaluronidases), *ebpA*, *ebpB*, and *ebpC* for adhesion, the *cps* gene cluster (*cpsA*–*cpsK*) for biofilm formation, *ace* (adhesin to collagen of *E. faecalis*) and *gelE* (gelatinase) for potential tissue damage. Additionally, components of the aggregation substance protein Asc10 was detected (*prgB/asc10, EF0149, asa1, EF0485*), involved in the initial attachment and infectivity of *E. faecalis*. Also, the *fsr* quorum-sensing system (*fsrA*, *fsrB*, *fsrC*) suggests the strain can regulate virulence factor expression in response to inflammatory signals. These findings highlight a genetic disposition that may be relevant for the strains capability of interacting with or modify disease severity.

Regarding antimicrobial resistance genes, a search against the Comprehensive Antimicrobial Resistance Database (CARD) (https://card.mcmaster.ca/) identified multiple antimicrobial resistance genes, including *dfrE*, *emeA*, *lsaA*, the *vanT* gene within the *vanG* cluster, and *efrA*. These genes encode resistance mechanisms such as trimethoprim-resistant dihydrofolate reductase (*dfrE*), multidrug efflux pumps (*emeA*, *efrA*), and vancomycin resistance (*vanT*) (Supplementary Fig. S1). The presence of these genes may simply reflect the strain's origin from a UC patient exposed to the hospital environment; however, they may also influence its persistence and host interactions in the DSS colitis model.

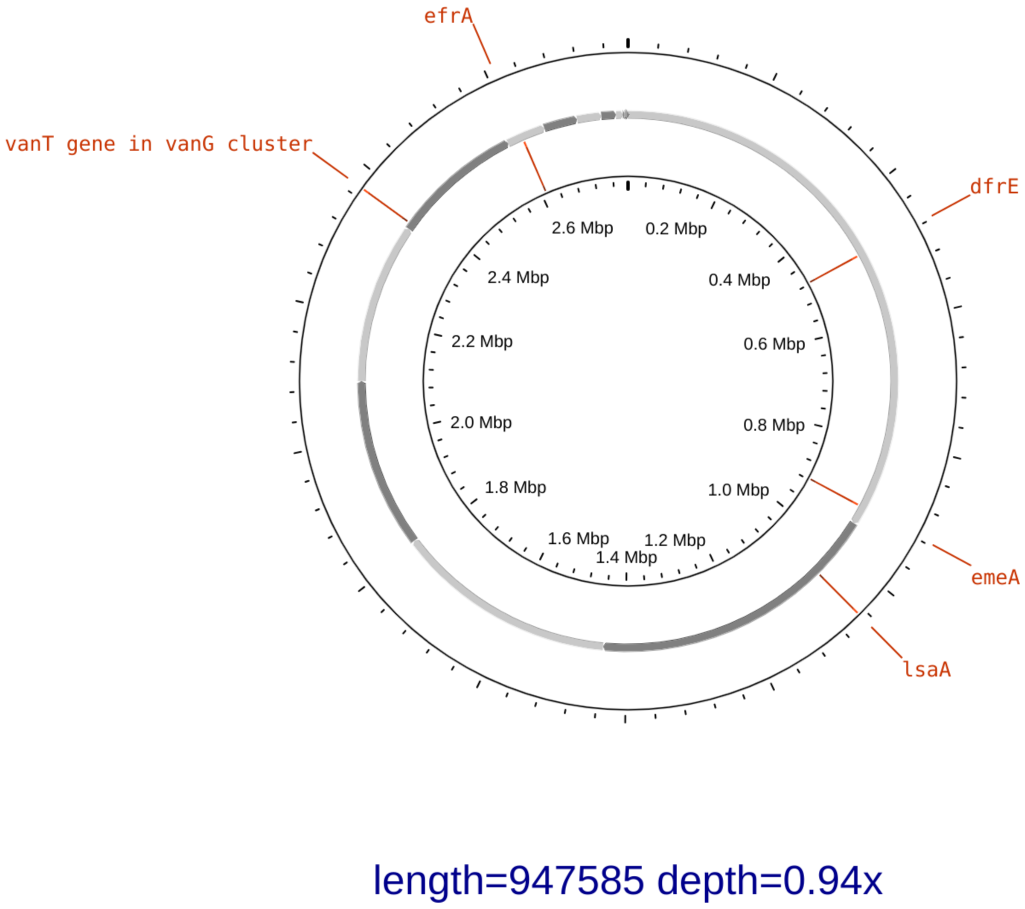

Supplementary Figure S1. Identified antimicrobial resistance genes in *E. faecalis* isolate used for monocolonization. Gene explanations: lsaA: lincosamide-streptogramin A; dfrE: trimethoprim-resistant dihydrofolate reductase; emeA: multidrug efflux pump protein belonging to the major facilitator superfamily; efrA: a component of the EfrAB efflux pump, belonging to the ATP-binding cassette transporter family; VanT: part of the VanG operon, which is associated with vancomycin resistance in enterococci.

Pilot Study: Optimal dose and scoring criteria in the DSS-induced colitis model

Induction of acute colitis in mice using dextran sulphate sodium (DSS) administration has been reported to have high variability in different mouse strains, between batches of DSS as well as dosage. Hence, in

the pilot study, we investigated the effects of different dosages of DSS in sterile drinking water (1.5%-3.0%) in age and gender matched conventional *versus* germ-free (GF) C57BL/6J mice. Following one week of acclimatization, mice were administered DSS in sterile drinking water over 7 days. During DSS administration, all mice were monitored daily with measurements of body weight, food intake, water intake and scoring according to disease activity. At day 7 of DSS, the mice were euthanized by heart puncture under isoflurane anesthesia followed by cervical dislocation and harvesting of organs (Supplementary Fig. S2). During the experiment, we also tested the suitability of a relevant scoring system for the model, assessing humane end points (HEP) (Supplementary Table S1). The HEP scoring system included the parameters weight loss, stool consistency, presence of visible bleeding, rectal prolapse and overall behavior. Mice were euthanized in cases of weight loss above 20% from DSS start, severe rectal prolapse, isolated behavior or reaching HEP score of 14 or higher.

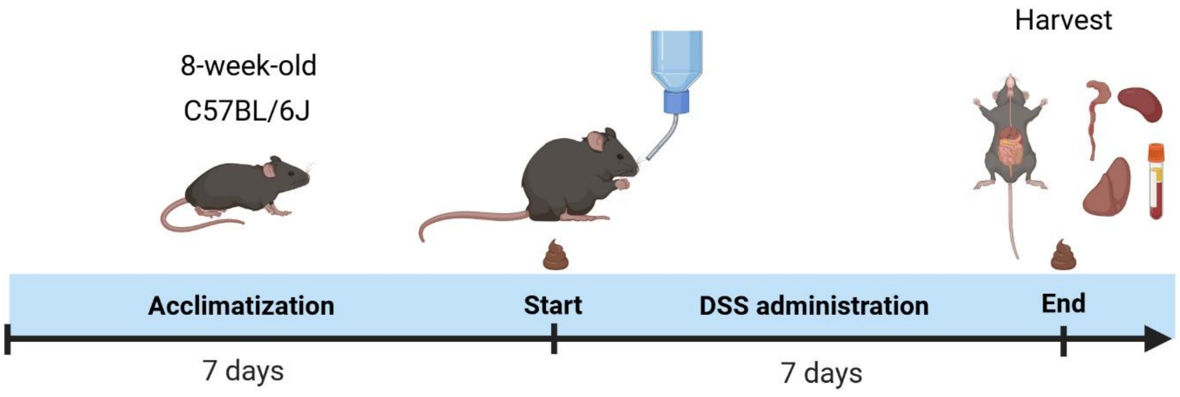

Supplementary Figure S2. DSS-induced colitis model study design.

**HEP scores**		**Score**
**Weight loss***	0-5 %	0
	5-10 %	2
	11-15 %	5
	16-20 %	7
	≥ 20 %	14
**Stool concistency**	Formed	0
	Loose, in face of WL < 15%	2
	Loose, in face of WL>=15%	4
	Gross diarrhea	6
**Bleeding**	No bleeding	0
	Mild bleeding, blood visible in stool or red colored rectum	1
	Moderate bleeding, visible from rectum/minimal visible in bedding/cage	3
	Severe bleeding, blood visible in stool or rectum, marked blood staining in the bedding/cage	6
**Rectal prolapse****	Absent	0
	Mild (1-2 mm protrusion)	1
	Moderate (2-3 mm protrusion)	4
	Severe (>3 mm protrusion+add)	14
**Behaviour**	Normal	0
	Mild piloerection or decreased activitiy/slight hunch back	3
	Significantly reduced activity and hunch back	7
	Isolated	14
**Total score**		

Supplementary Table S1: Humane endpoint (HEP) scoring sheet for DSS model and *E. faecalis*-monocolonization experiments. Evaluated daily during DSS administration. A total score of 14 was considered a HEP.

* Reference weight will be determined at "Day 0" (end of acclimation period, at start of DSS).

** Rectal prolapse is scored based on Pettan-Brewer & Treuting 2011 (PMID: 22953032).

Testing of different DSS concentrations were carried out in conventional and GF mice. GF C57BL/6J mice were obtained from the University of Bern Clean Mouse Facility. Conventionalized offspring from these GF animals, housed for more than two generations in a specific pathogen free (SPF) environment at the animal facility at Oslo University Hospital Rikshospitalet, Oslo, Norway were used in this pilot experiment together with GF mice that were housed in the isolator prior inclusion to the experiment. A total of 29 conventional C57BL/6J mice (15 males and 14 females, 8 weeks of age) were divided into 5 gender matched groups; controls (n=5), 1.5% DSS (n=6), 2.0% DSS (n=6), 2.5% DSS (n=6) and 3.0% DSS (n=6) (Supplementary Fig. S3a). A clear dose-dependent effect was observed as judged by per cent body weight (Supplementary Fig. S3d). Moreover, colon shortening was observed at DSS concentrations above 1.5% and histology score increased in a dose-dependent manner (Supplementary Fig. S3b and c). Circulating hemoglobin levels were slightly reduced at DSS doses 1.5% and 3.0%, otherwise unchanged compared to controls (Supplementary Fig. S3e). White blood cell counts were not changed at any of the DSS dosages tested (Supplementary Fig. S3f). Of note, HEP scores were relatively mild for all mice, ranging from 1-8 points out of 14 at day 7 after DSS administration (data not shown). Nevertheless, we observed visible immune cell infiltration, erosion and intestinal crypt damage on histology with increasing DSS dose as compared to controls (Supplementary Fig. S4).

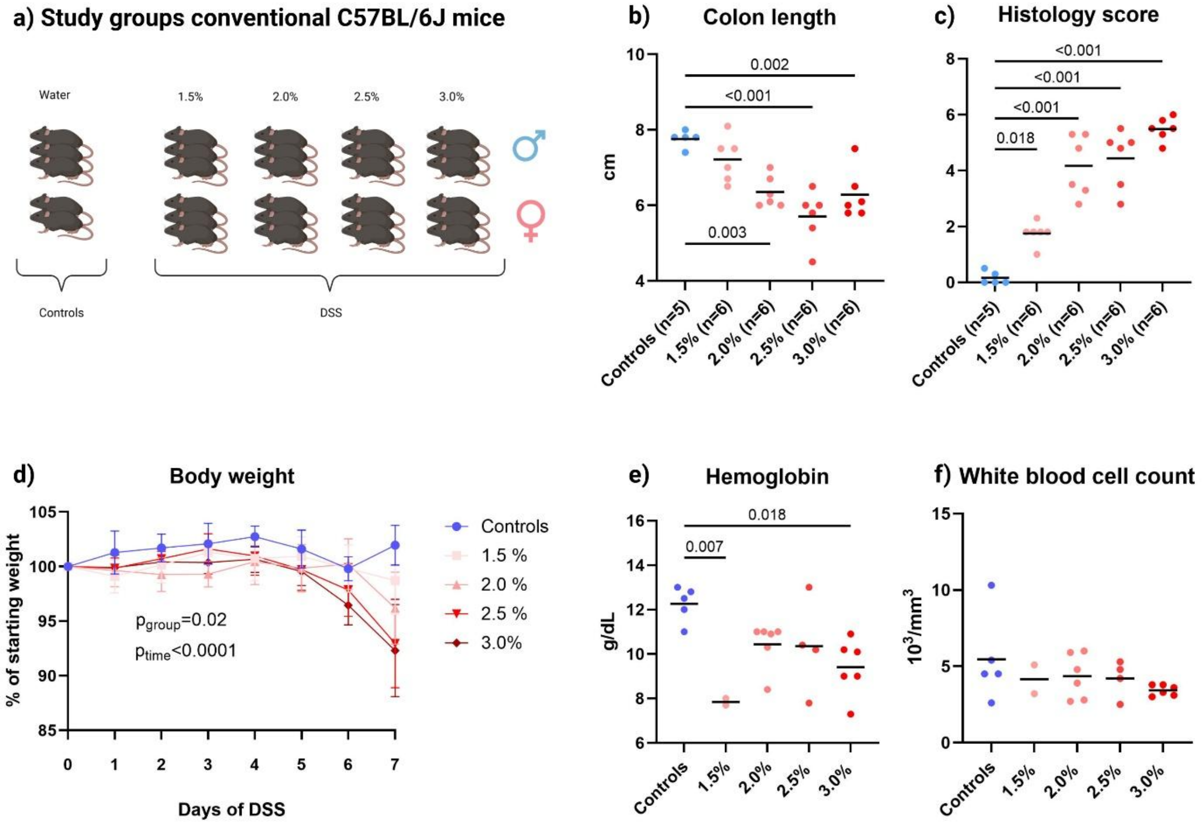

Supplementary Figure S3. Results from conventional C57BL/6J mice after 7 days of DSS administration. a) Group allocation for DSS dose testing. b) Colon length in cm. c) Histology scores calculated from whole colon sections. d) Body weight as percentage of starting weight. e) Hemoglobin levels measured in whole blood. f) White blood cell count. Data in b), c), e) and f) are shown as individual values with the mean indicated. Significant p-values from one-way ANOVA with correction for multiple comparisons are reported. Panel d) shows mean ± SD, with the p-values derived from a mixed-effects model (type III fixed-effect, sphericity not assumed), reported separately for group (column factor) and time.

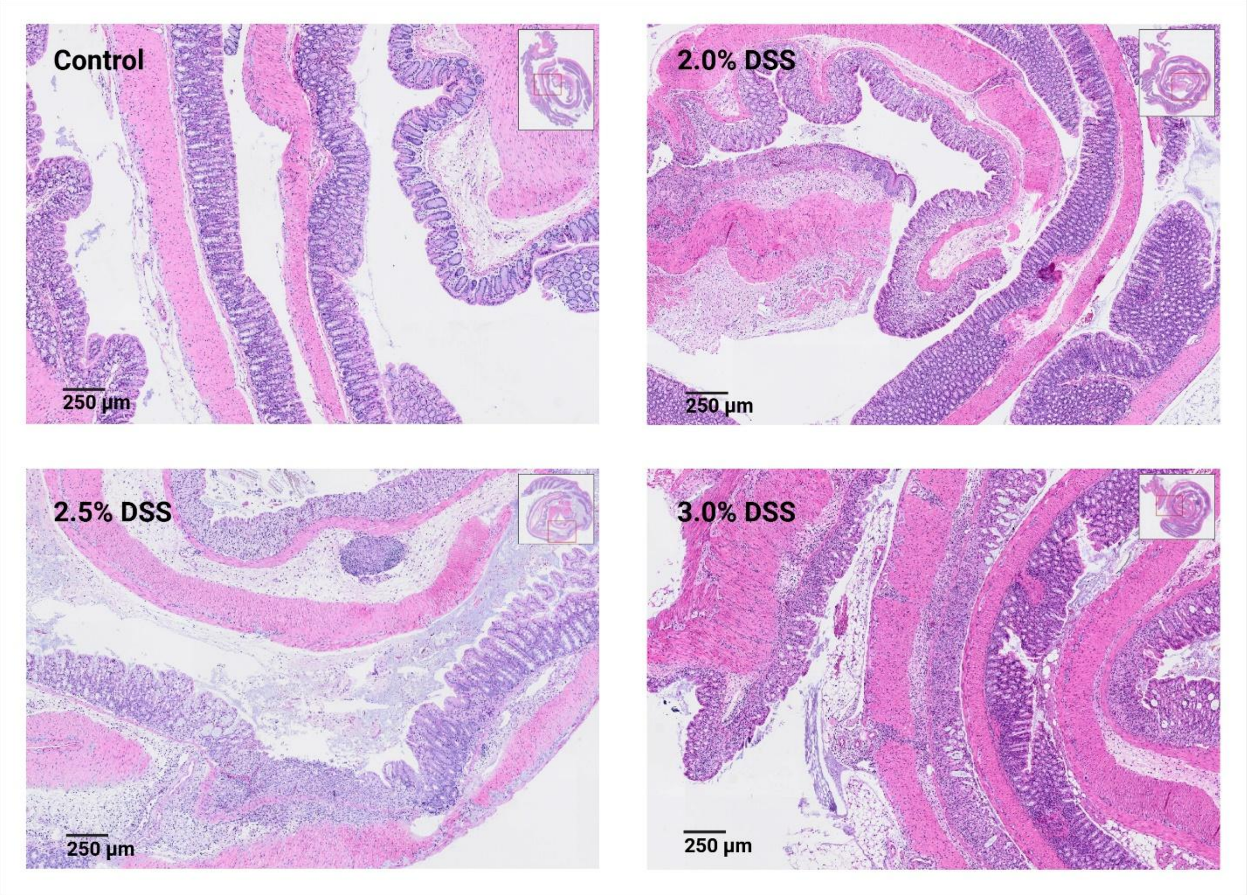

Supplementary Figure S4. Representative histology images from conventional mice after DSS treatment.

A total of 12 GF C57BL/6J mice were included in the initial pilot study batch (6 males and 6 females, age 8 weeks). The mice were divided into three different groups; controls (n=4), 2.0% DSS (n=4) and 3.0% DSS (n=4) (Supplementary Fig. S5a). All mice seemed to tolerate the DSS up to day 4 or 5. However, at day 5, 3.0% DSS was likely toxic to the mice, leading to massive bleeding despite modest weight loss (Supplementary Fig. S5d). As a result, three out of four mice were terminated at day 6 in the 3.0% DSS group due to reaching HEP. In a second batch of pilot study with GF animals (n=4), we confirmed that 2.5% DSS was tolerated by the mice (data from all GF mice are combined in Supplementary Fig. S5b-f). Similarly to observations in conventional mice, GF mouse colonic sections displayed a relatively normal crypt architecture in control mice (apart from very mild hyperplasia), with increasing infiltrations,

hyperplasia, edema, erosion and crypt loss at increasing DSS concentrations (Supplementary Fig. S6). However, sections from mice receiving 3.0% DSS supported the lower histology score with slightly less visible inflammation and intestinal damage than 2.0% and 2.5% DSS. The likely reason is that these mice were sampled at Day 6, one day earlier than the other groups.

In summary, these pilot experiments showed that, in our facility and with the described material and methods, 2.5% DSS seemed like a tolerable dose for robust colitis development in both conventional and GF C57BL/6J mice without reaching a premature HEP.

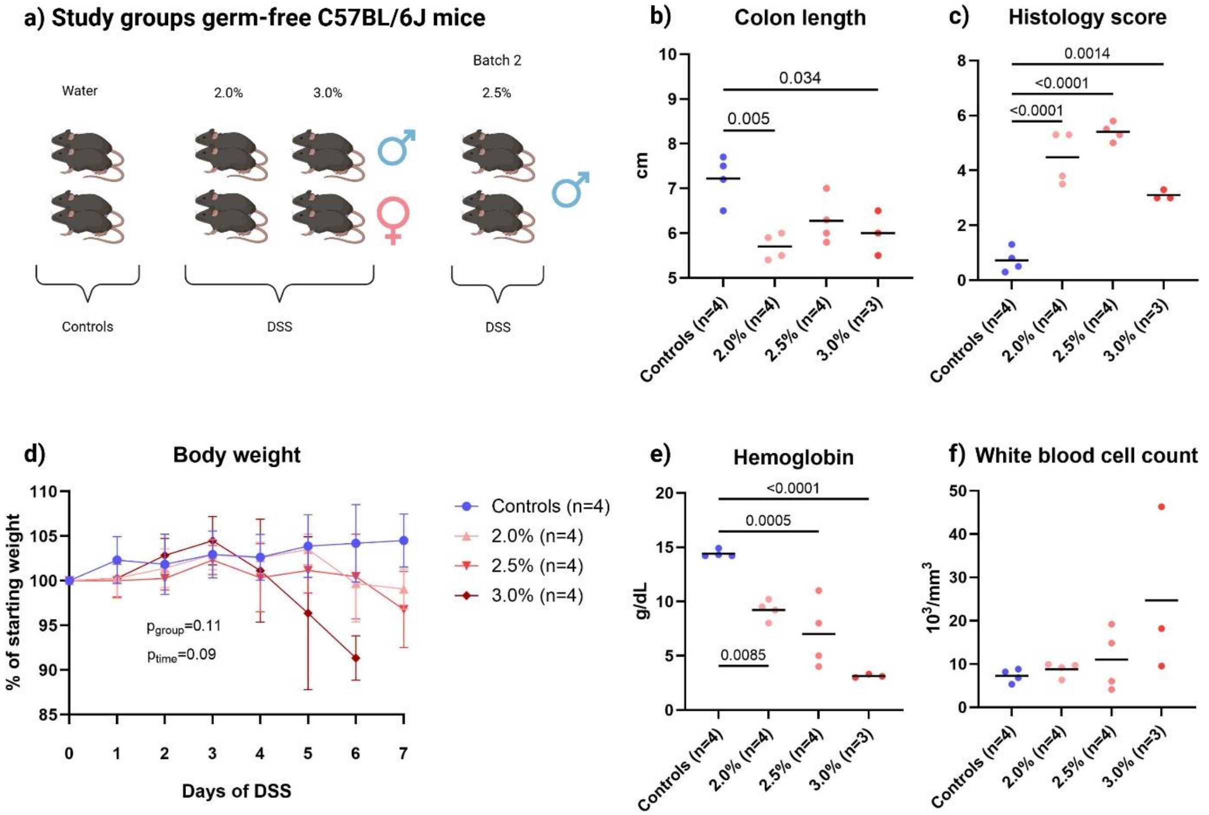

Supplementary Figure S5. Harvest measures from GF mice after 7 days of DSS administration. a) Group allocation for DSS dose testing. b) Colon length in cm. c) Histology scores calculated from whole colon sections. d) Body weight as percentage of starting weight. e) Hemoglobin levels measured in whole blood. f) White blood cell count. Data in b), c), e) and f) are shown as individual values with the mean indicated. Significant p-values from one-way ANOVA with correction for multiple comparisons are reported. Panel d) shows mean ± SD, with the p-values derived from a mixed-effects model (type III fixed-effect, sphericity not assumed), reported separately for group (column factor) and time.

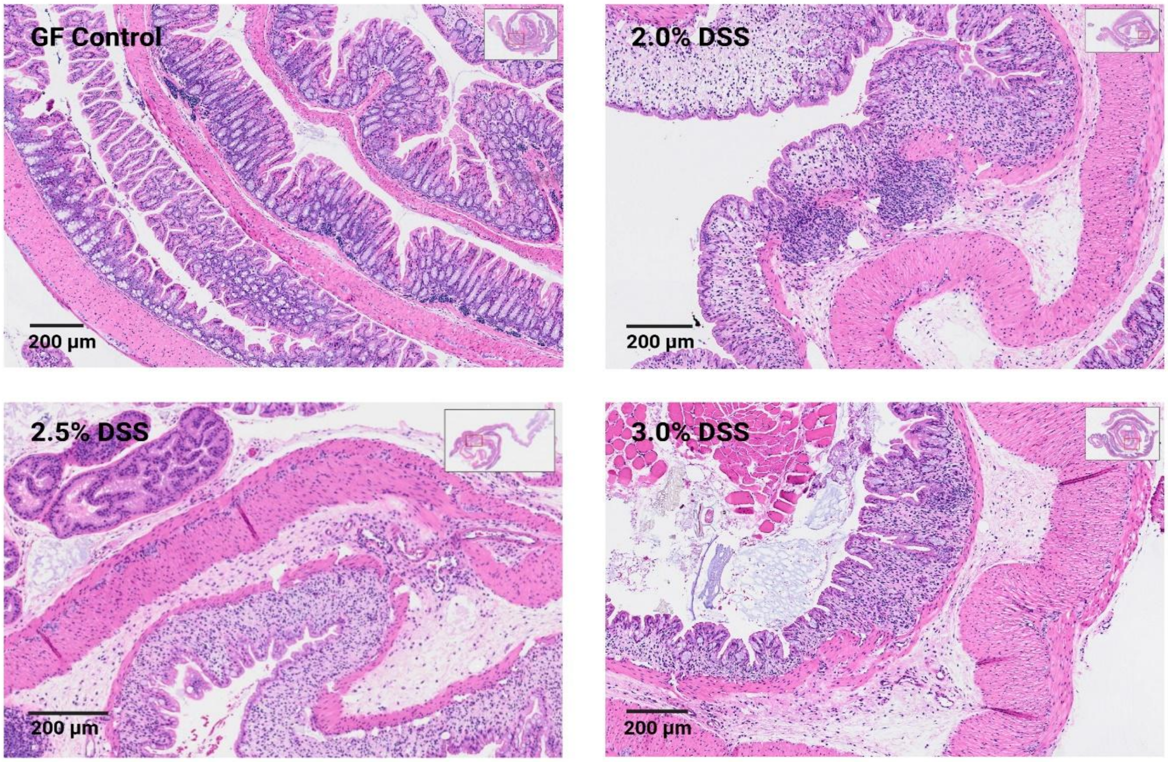

Supplementary Figure S6. Representative histology images from GF mice after DSS administration.

Sex as a biological variable

Both the pilot and colonization studies included mice of both sexes. To document the sex composition of the cohorts and assess potential sex-related influences, we performed exploratory sex-stratified analyses of body weight trajectories and terminal measurements (Supplementary Fig. S7). Detailed harvest data for all mice in the colonization study, including sex and body weight, are provided in the raw numerical dataset (https://zenodo.org/records/17829174). As some experimental groups contained small numbers of animals and an uneven distribution of males and females, these analyses are descriptive rather than inferential, and the study was not powered to detect sex-specific effects. Nevertheless, no consistent sex-associated differences were observed; body weight remained non-significantly different between DSS-treated groups when analyses were restricted to males ($p=0.75$, Supplementary Fig. S7c), consistent with the full dataset ($p=0.39$, main paper, Fig. 2a). Moreover, terminal body weight did not differ between males and females within both groups of colonized mice (with or without DSS) (Supplementary Fig. S7d-e).

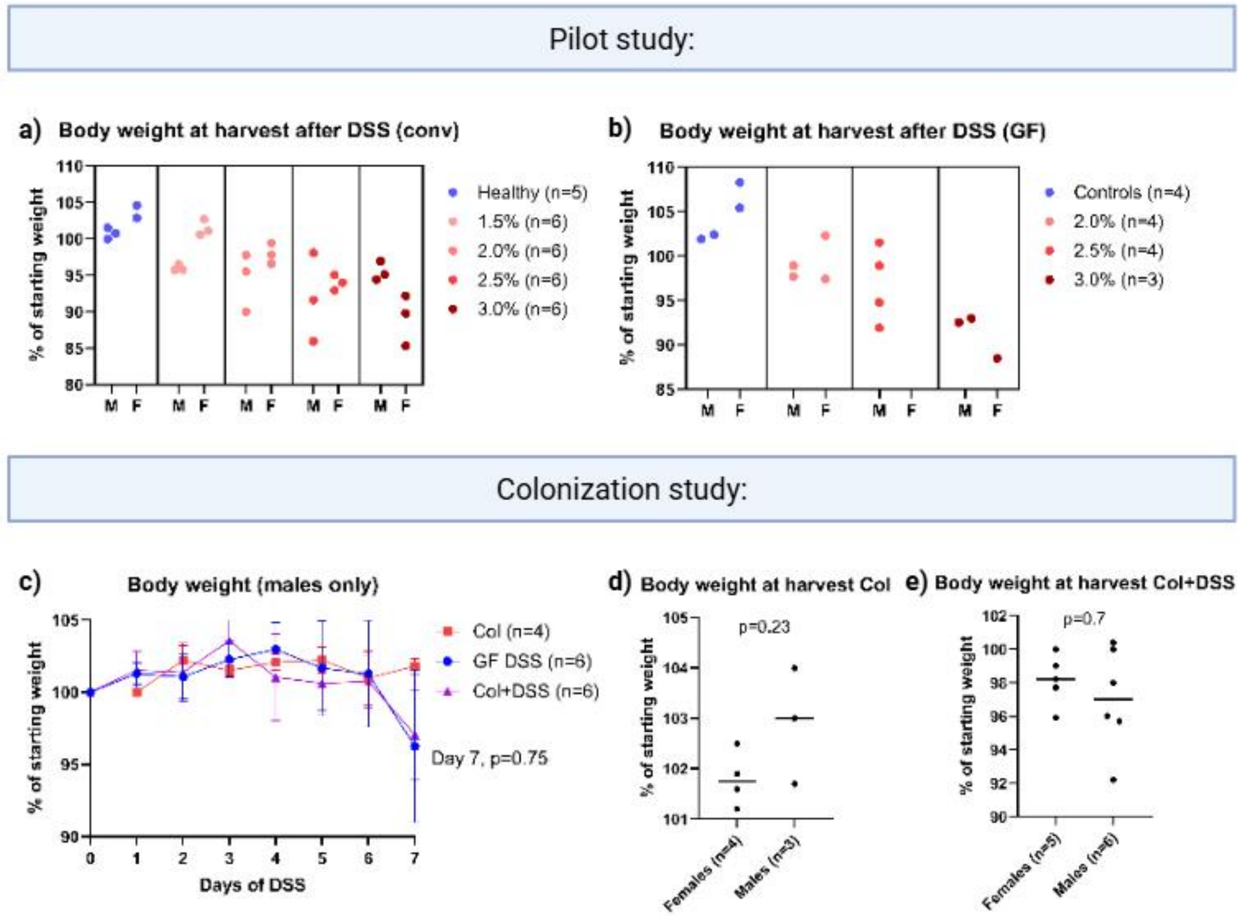

Supplementary Figure S7. Sex distribution and exploratory assessment of sex-related differences in body weight across pilot and colonization studies. a) Body weight at harvest in conventional (conv) mice exposed to different DSS concentrations in the pilot study. Individual mice are indicated by dots and separated by sex (M=male, F=female). b) Body weight at harvest in GF mice exposed to different DSS concentrations in the pilot study, separated by sex. c) Body weight curves during DSS exposure in the colonization study, showing male mice only; these data correspond Figure 2a in the main manuscript. Comparison between the two DSS-treated groups at Day 7 was performed using a two-tailed unpaired *t* test. Panel d) and e) show endpoint body weight differences between females and males for colonized mice without DSS (Col, n=7) and the monocolonization + DSS group (Col+DSS, n=11), respectively, with median indicated. P-values were generated using two-tailed Mann-Whitney *U* test.

Bacterial translocation to mesenteric lymph nodes at endpoint

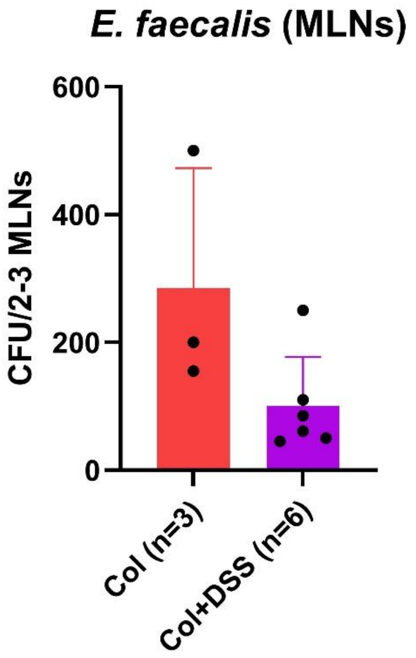

Supplementary Figure S8. Colony-forming unit (CFU) counts from aerobic overnight cultures of mesenteric lymph nodes (MLNs) collected at termination from colonized mice without DSS (Col, n=3) and colonized mice treated with DSS (Col+DSS, n=6). Each point represents one mouse; lines indicate median values with 95% CI. The difference between groups was assessed by Mann–Whitney *U* test.
